# Digital workflow for mini-implant–assisted rapid palatal expander fabrication–a case report

**DOI:** 10.1186/s12903-023-03589-5

**Published:** 2023-11-20

**Authors:** Li-Fang Hsu, Won Moon, Shih-Chin Chen, Kelvin Wen-Chung Chang

**Affiliations:** 1 Department of Dentistry, National Taiwan University Hospital, Hsinchu Branch, Hsinchu, Taiwan; 2 Graduate Institute of Clinical Dentistry, School of Dentistry, National Taiwan University, Taipei, Taiwan; 3 The Forsyth Institute, Cambridge, MA USA; 4 Ajou University School of Medicine, Suwon-si, Korea; 5 Section of Orthodontics, School of Dentistry, Center for Health Science, University of California, Los Angeles, CA USA; 6 Breeze Dental Center, No. 588, Xianzheng 2Nd Rd., Zhubei City, Hsinchu County Taiwan (R.O.C.)

**Keywords:** MSE, MARPE, Maxillary expansion, Digital workflow

## Abstract

**Background:**

Non-surgical mini-implant assisted rapid palatal expansion, or midfacial skeletal expansion, is a paradigm-shifting concept that in recent years has expanded the envelope of orthopedic movement in the transverse direction for adult patients. Although adding mini-screws to a rapid palatal expander is not complicated, accurate and successful expansion strongly depends on the device’s position and its relation to the resisting structures of the maxillofacial complex.

**Case presentation:**

This article presents a digital workflow to locate the optimal position of the Midfacial Skeletal Expander (MSE) device in a CBCT-combined intraoral scan file and describes how to transfer the MSE position intra-orally with properly sized bands during the device fabrication. The complete digital workflow of MSE fabrication and its application for a Class III orthognathic surgical case is presented in detail.

**Conclusions:**

This report describes a completely digital process that can accurately position the MSE device according to the orientation and morphology of maxillary basal bone, which is crucial in adult cases demand maxillary expansion.

## Background

Rapid palatal expansion (RPE) is an effective approach for treating constricted maxilla or transverse deficiency in children and young adolescents. It was first described more than 100 years ago as a tooth-borne device and then further developed in diverse forms: banded and bonded maxillary expanders [1–4], as well as removable expanders, such as the Schwarz expander [5, 6]. RPE’s treatment effects have also been thoroughly investigated [7–9]. Depending on varying degrees of mid-palatal sutural interdigitation and resistance from other peri-maxillary sutures, maxillary basal bone expansion (skeletal component) accounted for only 20%–50% of the total expansion, for both a Haas type of expander and other types of RPE [10, 11]. Furthermore, the expansion results have a substantial degree of relapse, so an overcorrection and a long retention period for bone consolidation are critical with the traditional tooth-borne RPE appliances [11–14].

The concept of mini-screw–assisted rapid palatal expansion/ midfacial skeletal expansion (MARPE/ MSE) emerged more than a decade ago [15–18]. In the MARPE/MSE designs, mini-screws substitute teeth as the main anchorage receiving the expansion force and transfer the force to the underlying skeletal structures and hold the positions of the two expanded maxillary halves during the bony bridging of the two segments. After a skeletal expansion, the compensated teeth can be decompensated and aligned into a more physiological position. The reported basal bone expansion results of various MARPE designs range from 40%–95% in adult patients [18–20]. This wide range in skeletal components can be attributed to various MARPE designs that produce different results. Some are true bone-borne devices, but some are hybrid devices with significant tooth movement. The MSE has two banded teeth incorporated in its design to stabilize the jackscrew position during the expansion, but the arms attaching the bands to the jackscrew are a soft alloy and the expansion force transferred to the anchor teeth is minimal. The force vector and magnitude generated by each MARPE also affect the pattern and quality of expansion. When MARPE is used properly, it can produce a breakthrough result in regard to both patient age and treatment efficacy.

However, the MARPE method is technique-sensitive, and the position of the mini-screws can have a significant effect on the success rate and the pattern of expansion of such devices. Maxillary bone thickness [21] and the surrounding anatomical structures should be carefully examined before mini-screw placement. Before deciding on which MARPE to use, one must consider the two competing concepts: bone-driven system vs. resistance-driven system. The bone-driven system favors the position of the min-implant to be in the area of a large bony mass for stability, usually in the anterior palate. This placement generates an anterior force vector which produces a “V-shaped” expansion with limited posterior skeletal expansion [22, 23]. MSE is a resistance-driven system in which the force vectors are generated directly against the resisting structures, which are mostly in the posterior region. The posterior force vector is necessary for achieving a parallel expansion with a good split of the posterior nasal spine [24]. However, the posterior palatal bone is thinner than the anterior region, and the mini-implants must be placed immediately lateral to the mid-palatal suture where the bone density and volume are greater [25]. A bicortical engagement of mini-implants is an essential part of MSE placement, to maximize the skeletal component of expansion and to reduce implant failure [26]. Overlooking the mini-implant position may result in negative sequelae, such as penetration of the mini-screw across the mid-palatal suture, engaging the septal structure, monocortical engagement, tilting of the devices, and irritation of the inferior nasal conchae. Li’s research examined whether bi-cortical engagement or anteroposterior positioning of the MSE affected the success rate and found that both bi-cortical engagement and posterior positioning of the MSE resulted in better expansion results [26]. Poorly positioned mini-screws or overlooked anatomical factors such as a skewed maxillary suture or canted palatal vault, can produce unsatisfactory expansion results [24, 27]. On the other hand, if mini-screws on a single side fail due to inadequate positioning, then the appliance will become tooth-borne on the failed-screw side. This will result in a detrimental effect on the banded first molar on that side and often leads to the expansion failure.

To tackle this problem, recent publications have introduced digitally aided design processes [28–30]. Cantarella et al. used commercially available software to establish an optimal MSE position first and then printed out the position guide for soldering on a stone model [28]. Giudice et al. used Dolphin software to import a negative MSE template to position the expander and the mini-screws, then printed the maxillary model combined with a negative MSE template for soldering [29]. These two methods demonstrated the concept of virtual mini-screw placement. However, both approaches still require a band selection and pick-up impression appointment since bands are still needed to generate the stone model for soldering. Hence, the process is not fully digital.

This article aims to describe a complete digital solution, from mini-screw positioning to accurate molar band selection, using a combination of various commercial software for Midfacial skeletal expander II (MSE II, BioMaterials Korea, Korea). The digital workflow is further demonstrated in a case report of a Class III orthognathic surgical case.

## Digital workflow

### Software

Autodesk Meshmixer (Autodesk Inc., San Rafael, CA, USA), 3Shape Implant Studio (3Shape, Copenhagen, Denmark), 3Shape Dental System (3Shape, Copenhagen, Denmark).

### Prepared materials

**Fig. 1:**
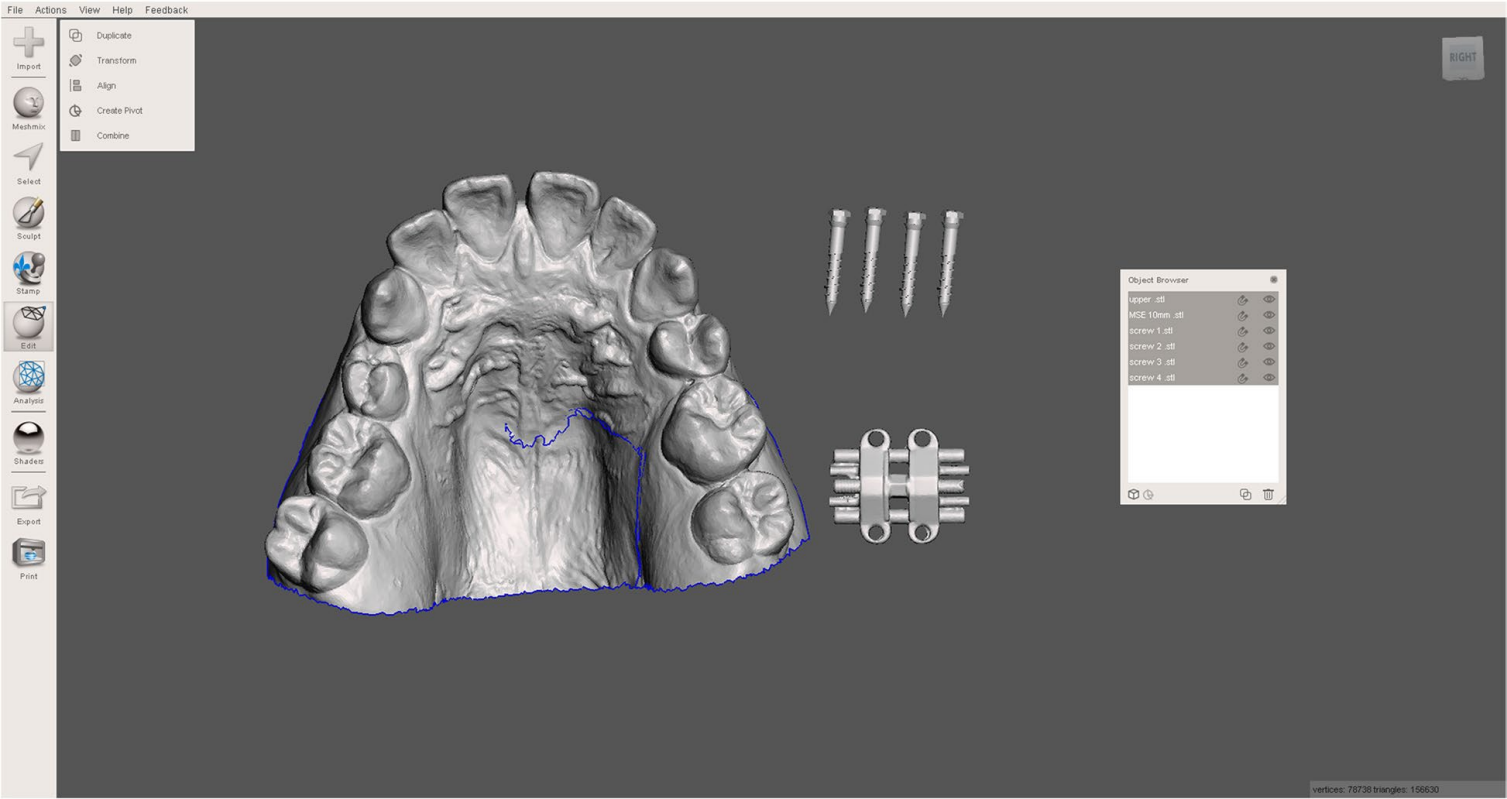
Scanned surfaces of dental model, mini-screw, and MSE device

Digital model of the upper arch, cone beam computed tomography (CBCT) image of the maxilla, MSE screw in the STL file (acquired by reverse engineering), MSE II expander in the STL file (obtained from the desktop scanner) (Fig. 1).

### Operation procedures

#### Step 1. Preliminary placement of the MSE

**Fig. 2:**
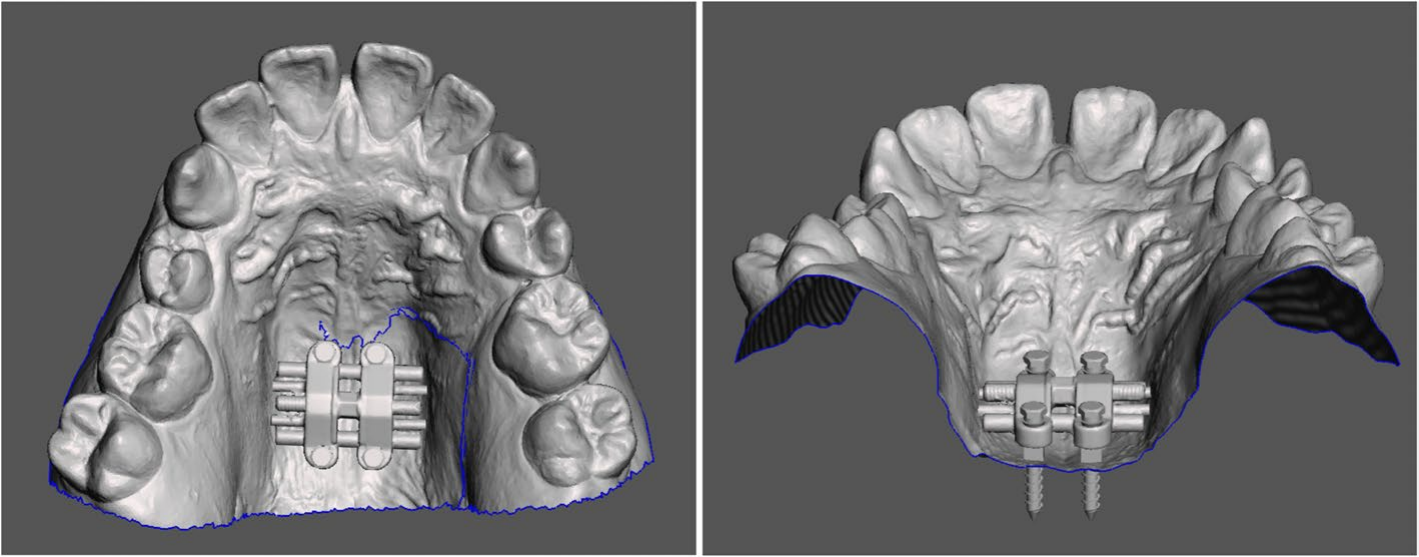
Merged multiple surfaces and positioning

All the information in STL format can be imported and moved freely in the open-sourced Autodesk Meshmixer software. The MSE expander and four screws are preliminarily placed according to the anatomical structures and clinical preference. The distance of the MSE body to the palatal roof is also examined, which should be as close as possible. Next, the MSE expander, four screws, and dental model are combined and restored as a single file, which is then exported to 3Shape Implant Studio software (Fig. 2).

#### Step 2. Verification of MSE position

**Fig. 3:**
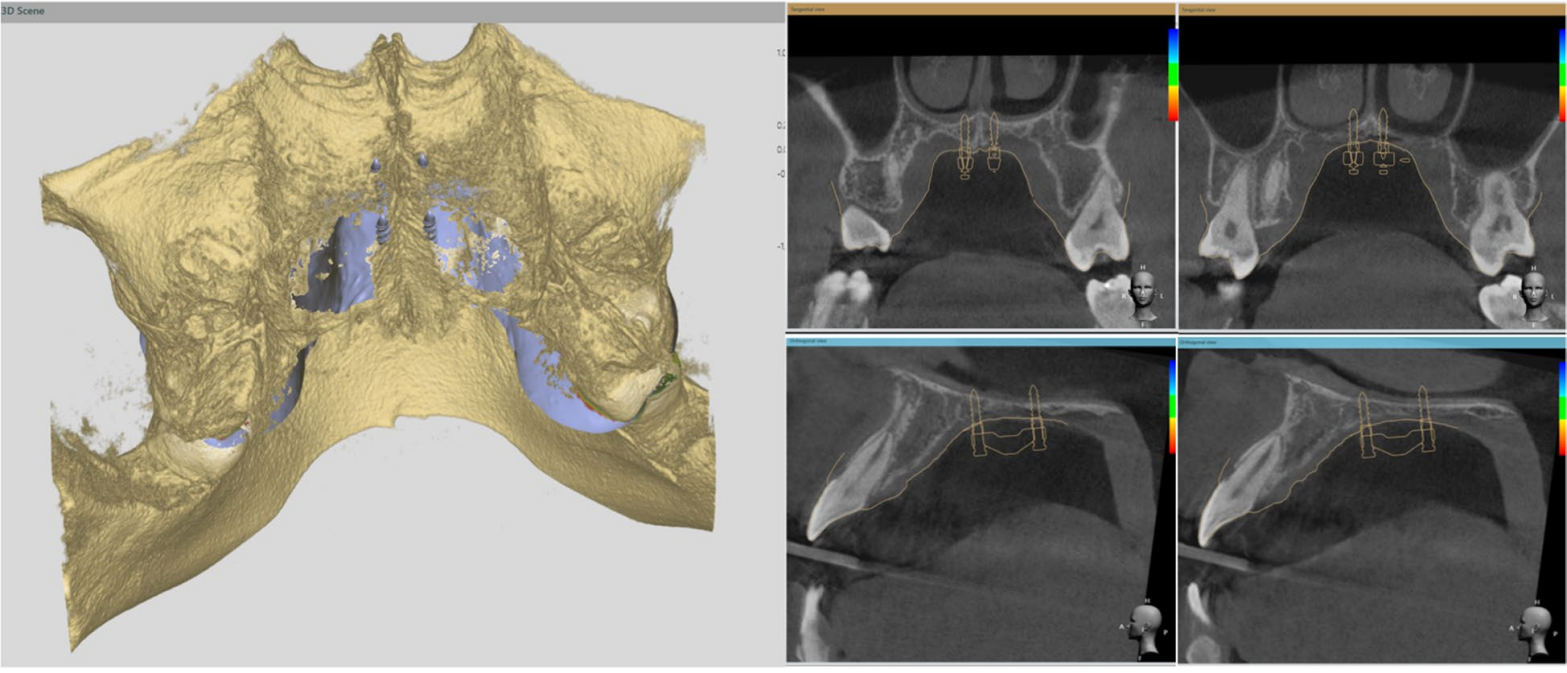
Importing merged surface with CBCT and verification of appliance position and determination of the appropriate length of the mini-screws. The actual distance from the inferior surface of MSE to the superior cortical plate of the maxilla can be measured, which were 9.51 mm (anterior) and 6.97 mm (posterior). Therefore, 11 mm for the anterior and 9 mm for the posterior screws were chosen to achieve bicortical engagement

In 3Shape Implant Studio, the CBCT image is imported and integrated with the model from step 1. The MSE and mini-implant position can be verified if their relations to the anatomic structures (mid-palatal suture, septal structure, palatal plate, zygomatic buttress bones, inferior nasal conchae, and nasal cortical layer) are appropriate (Fig. 3).

Otherwise, we must return to step 1 to modify the MSE position and then proceed to step 2 to verify again. Once the MSE position is confirmed, between the zygomatic buttress bones with bicortical engagements, the distance from the MSE to the nasal floor is measured, and the length of the mini-screws is determined accordingly.

#### Step 3. Segmentation of U6

**Fig. 4:**
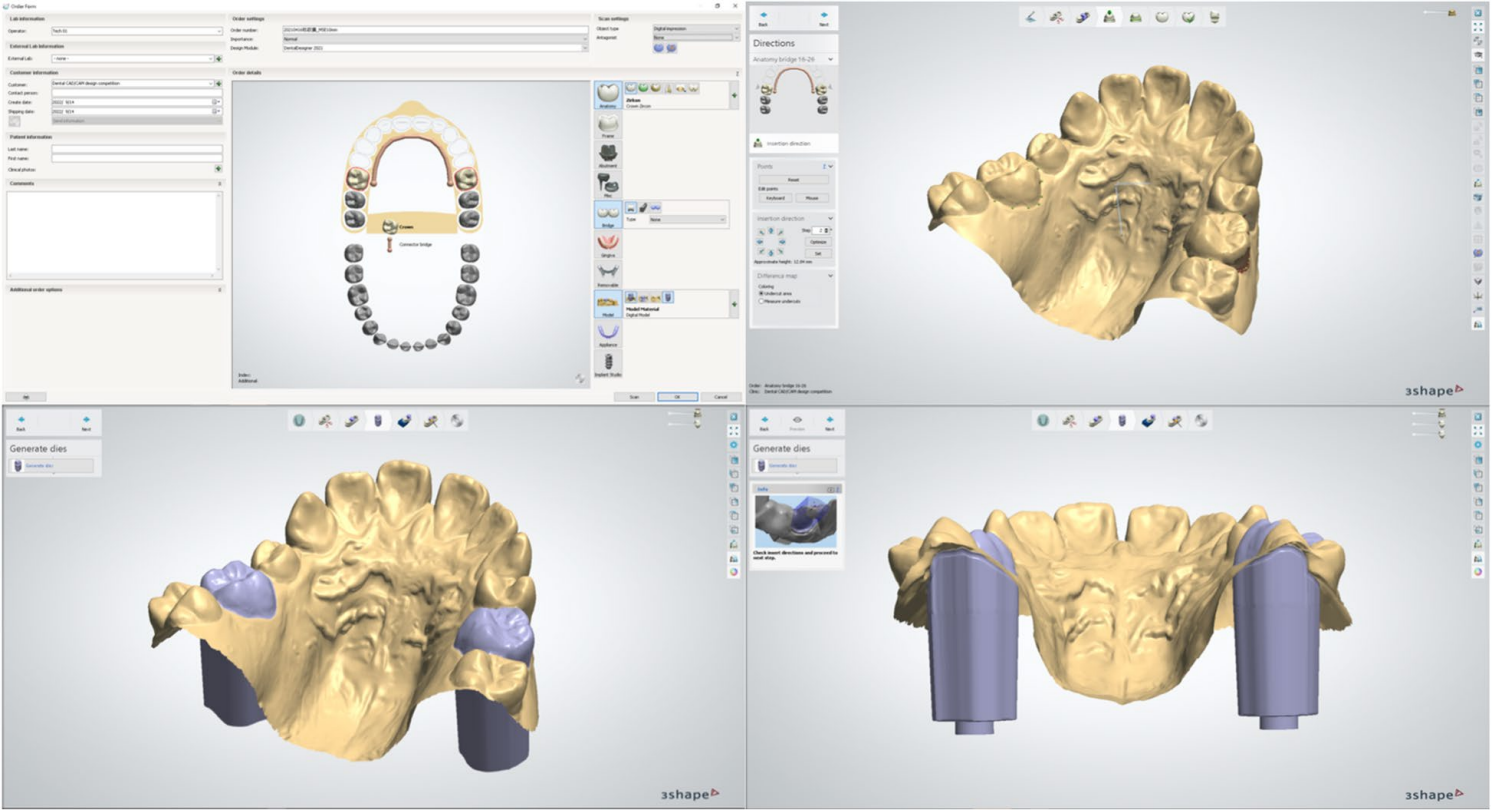
Upper first molar segmentation process in software. The insertion path can become parallel when the 16–26 bridge mode was chosen

We use the 3Shape Dental System’s software, model builder mode, to isolate the upper first molars from the upper dentition. The bridge mode can be applied to ensure parallel paths of insertion for these two molars (Fig. 4).

#### Step 4. Virtual integration of all information

Combining the step 3 model with that of step 1, the MSE, maxillary model, and first molar models are integrated into the software. The model is then exported to Meshmixer software for the design of the transfer index.

#### Step 5. MSE transfer index design and model printing

**Fig. 5:**
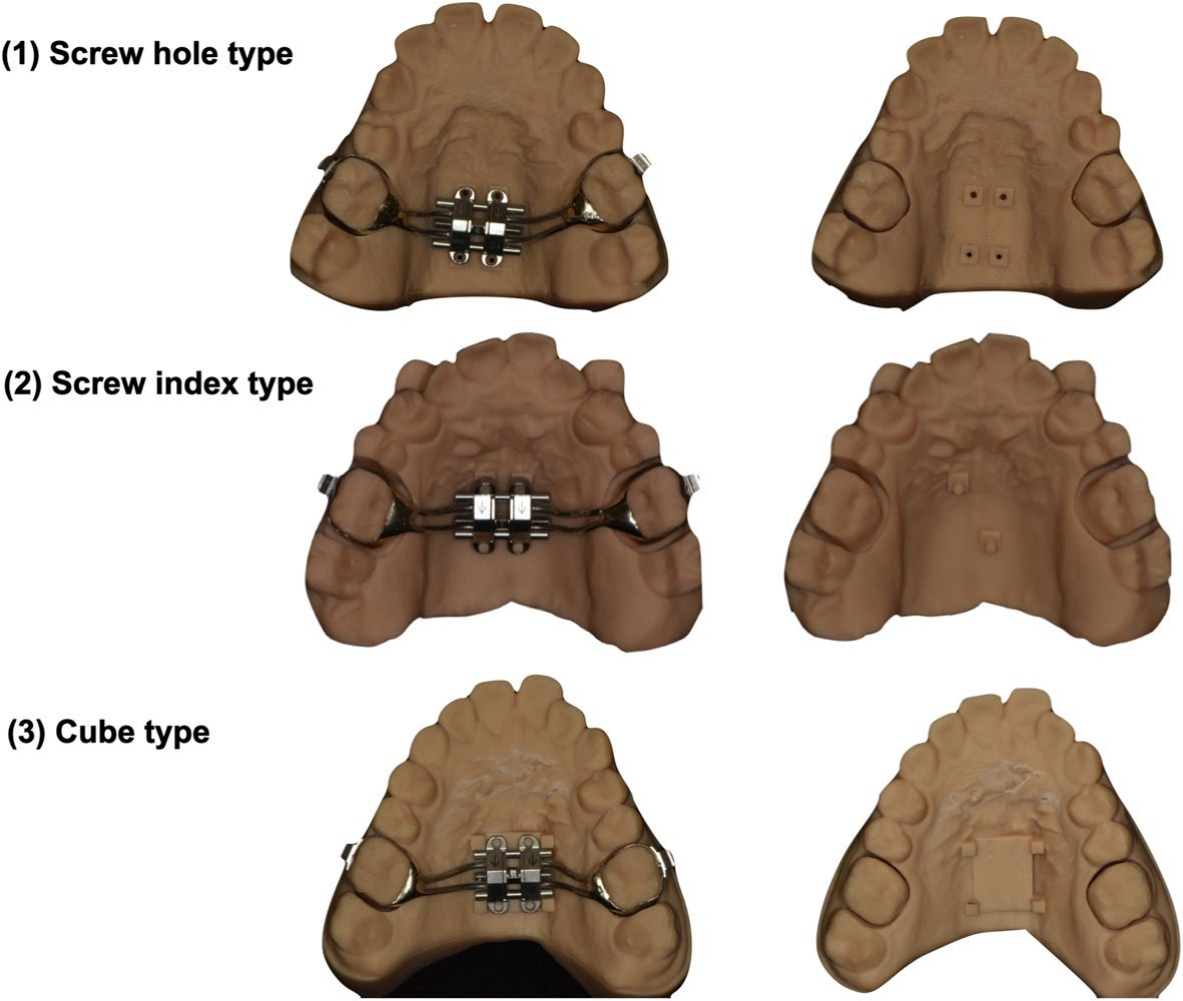
The transfer index can be designed to connect the expander with the upper arch model. Three types of MSE transfer indices can be used through similar designing workflows

Meshmixer offers several ways to design the transfer index (Fig. 5): (1) the “screw hole” type, the internal threads were built in the 3DP resin model in advance. Therefore, the MSE expander can be placed and connected securely and tightly with the 3DP resin model by the real mini-screws.; (2) the “screw index” type, The MSE expander can be connected with the 3DP resin model by the screw indices. Usually, two indices diagonally are good enough for positioning. But the thin screw index is easy to break when the insertion pathway of the expander screw is not parallel with the insertion pathway of two molar bands; (3) the “cube type”, the position of the MSE expander can be marked by some cubes around its’ corners. This is the easiest and most durable way among these three designs. Four cubes can be designed around the corners of the expander to secure its position; and for the vertical position of the expander, four cubes or a plane of a specific heights can be designed to transfer the vertical position of MSE precisely.

When the final step of this virtual design is complete, a resin model with the expander transfer indices and the two removable upper first molars is fabricated by 3D printing (Fig. 5).

#### Step 6. Selection of U6 band on the 3DP model

The appropriate bands are easily selected and precisely seated to the 3DP model.

#### Step 7. Combination of bands with MSE bars

**Fig. 6:**
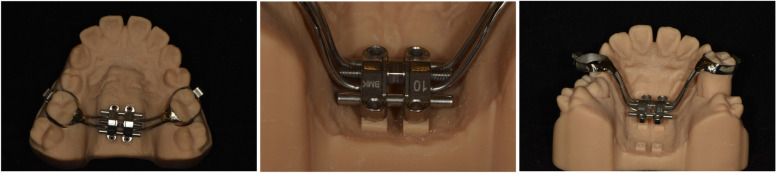
The selected bands were soldered with MSE bars. The whole device has only one path of insertion because of the parallel molar models. The MSE position was transferred accurately from the virtual design to the physical resin model

The U6 bands and the MSE bars are soldered, and their relative positions are maintained. The planned MSE position on the software is accurately transferred to the physical 3DP resin model (Fig. 6).

## Case presentation

**Fig. 7:**
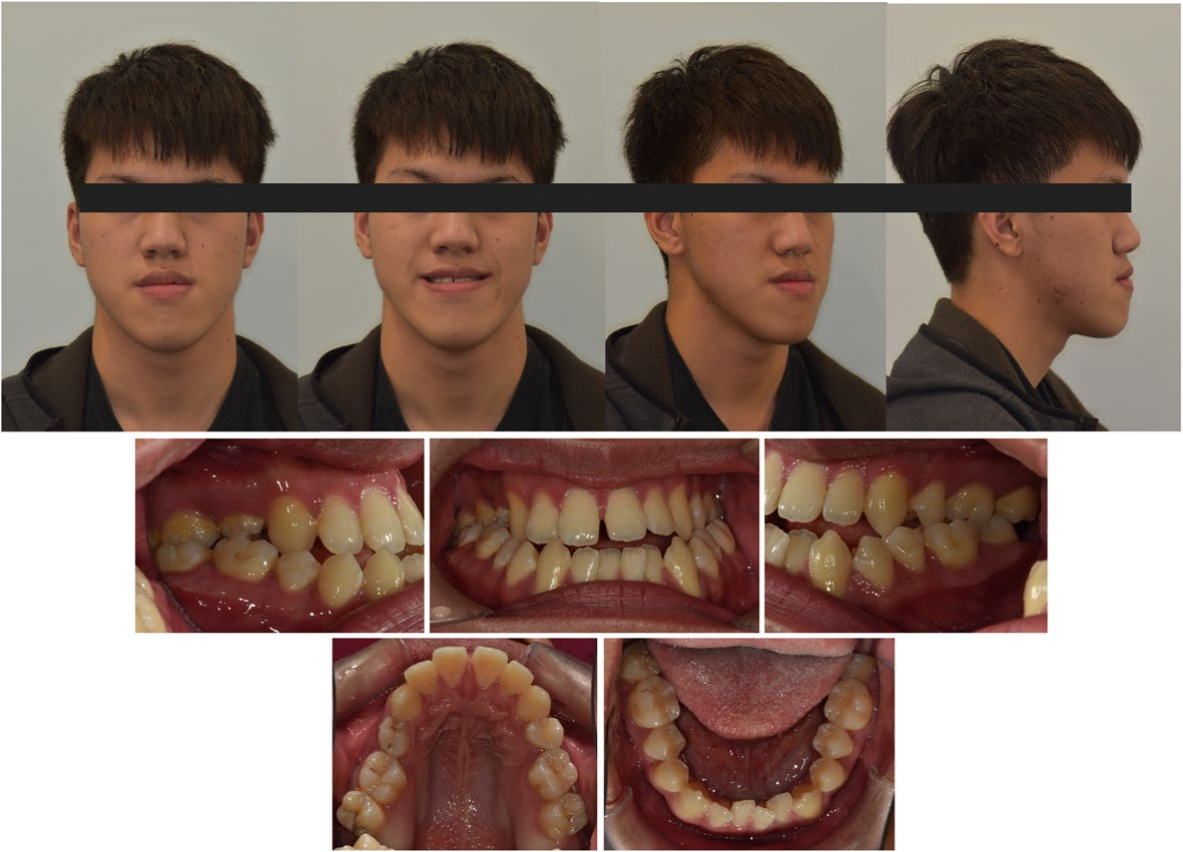
Initial extraoral and intraoral photo records

A 17-year-5-month-old male patient came for a consultation with chief complaints of diastema, facial asymmetry, and chin prognathism. Clinically, the patient had apparent facial asymmetry with a larger volume on his right side. His chin deviated to the left side, and his right side downward occlusal plane canting was also evident. The lateral profile revealed a retrognathic maxilla with an excessive lower jaw projection (Fig. 7).

An intraoral examination revealed a bilateral Class III canine and molar relationship with anterior open-bite. The overjet was -5 mm, and the overbite was -2 mm. Despite the severe dental compensation, the posterior teeth were still in a bilateral crossbite relationship. The upper 2^nd^ premolars were missing (Fig. 7).

**Fig. 8:**
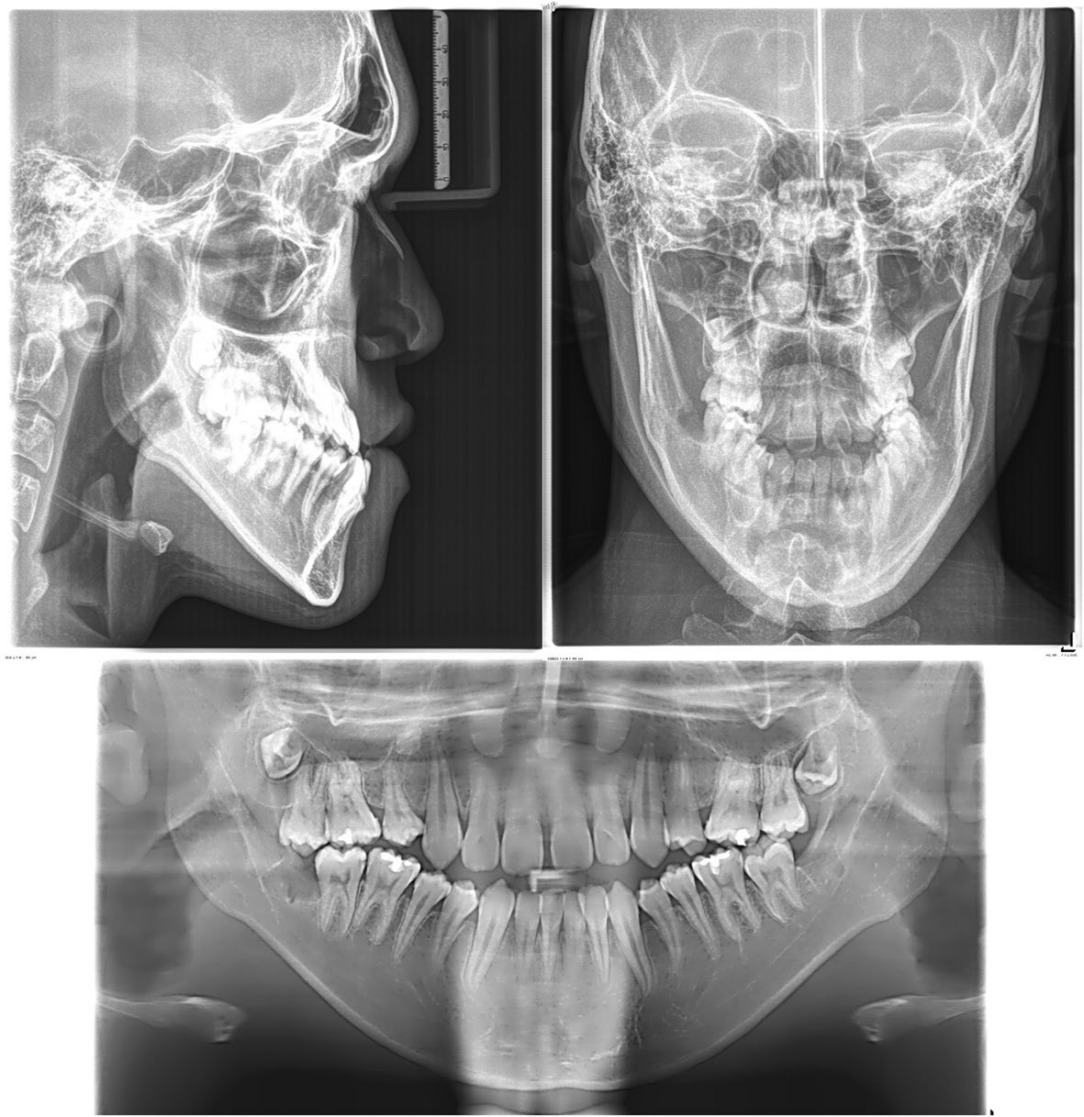
Initial lateral cephalometric, posteroanterior cephalometric, and panoramic radiographs

The panoramic radiograph showed a normal periodontal status with recently extracted lower third molar sockets. Regarding a cephalometric analysis, the patient had a skeletal Class III relationship with a hyperdivergent facial pattern, flared upper incisors, and retroclined lower incisors (Fig. 8).

## Treatment objective

The treatment objective was to correct the facial asymmetry and Class III skeletal pattern through a surgical treatment: maxillary advancement and mandibular setback. For the transverse discrepancy, maxillary skeletal expansion was planned to avoid a three-piece maxillary surgical procedure.

## Treatment progress

Before the treatment, a cone beam computed tomography and intraoral scans were performed to acquire the data needed for the MSE digital workflow. Following steps 1 through 5, a model with the MSE transfer indices was designed and 3D printed. The isolated upper molar crowns in a parallel orientation were sized for bands and they were soldered.

**Fig. 9:**
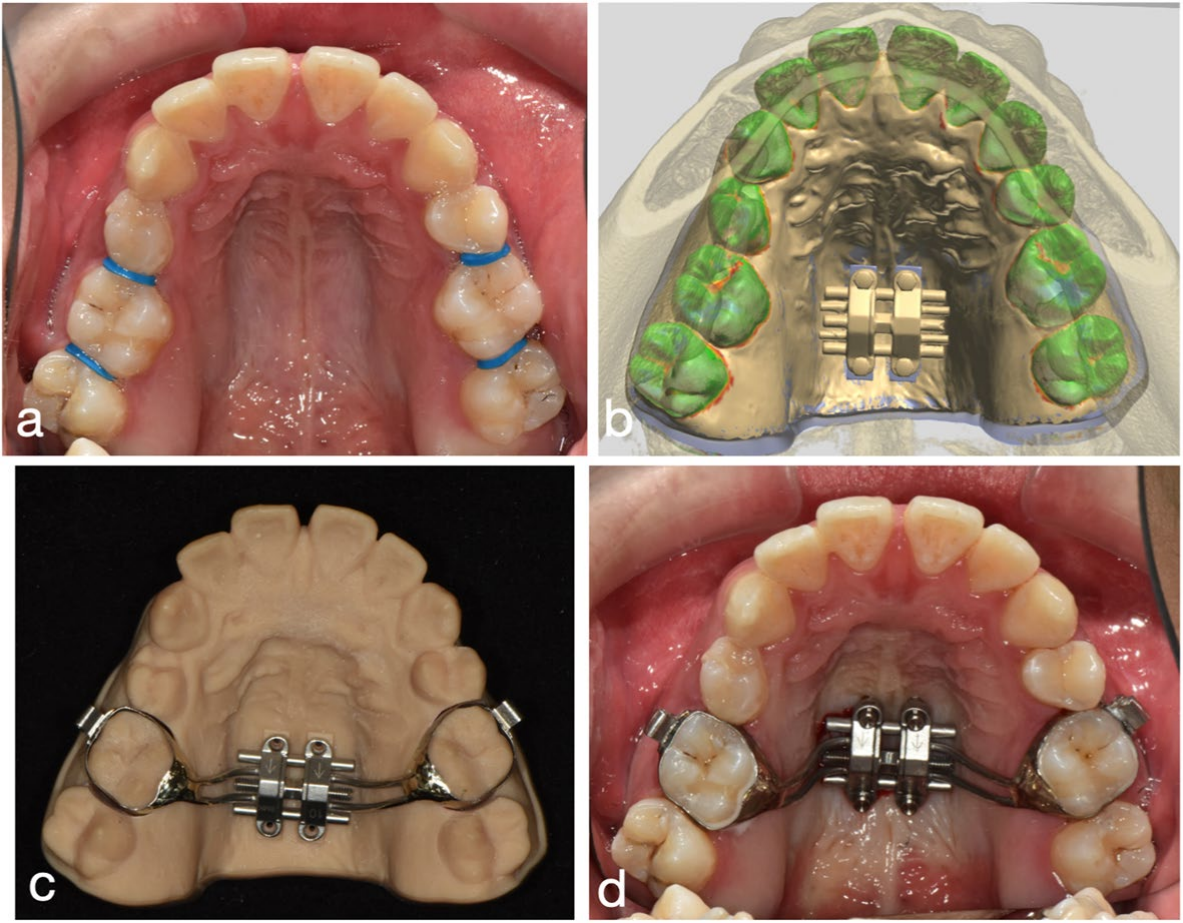
Comparison of the MSE position. a. The separators were placed one week before the MSE insertion date. b. on the virtual plan, a 10 mm MSE was placed. c. exported resin model, d. the final position in the patient’s mouth. Though the MSE seemed to be slightly shifted in the patient’s mouth, it was aligned to the bony maxillary suture

The patient came to the clinic one week before the MSE insertion date for molar separator application (Fig. 9a).

**Fig. 10:**
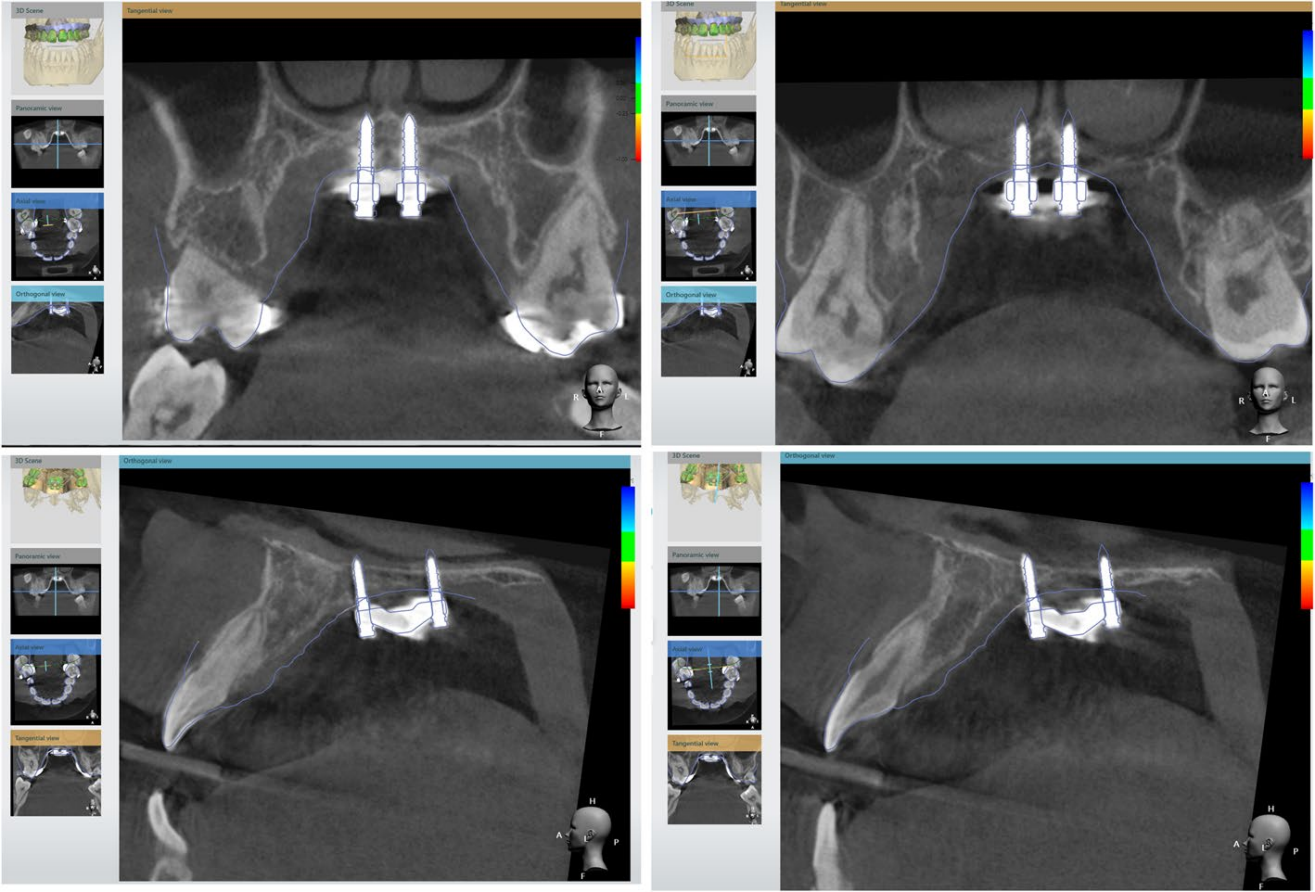
After MSE insertion, CBCT further proved predictability of the digital workflow. The distance of bilateral mini-screws to the mid-palatal suture were precisely the same for both anteriorly and posteriorly

On the day arranged for the MSE delivery, the bands were well-fitted, with the appliance in a proper position. The four mini-implants were inserted (Fig. 9b,c and d). According to the maxillary height measured via the merged dental and CT file, 1.8 × 11 mm anterior screws and 1.8 × 9 mm posterior screws were chosen for this patient. A CBCT taken immediately after insertion verified an accurate MSE position as a virtually designed and bi-cortical engagement of all four mini-implants (Fig. 10).

**Fig. 11:**
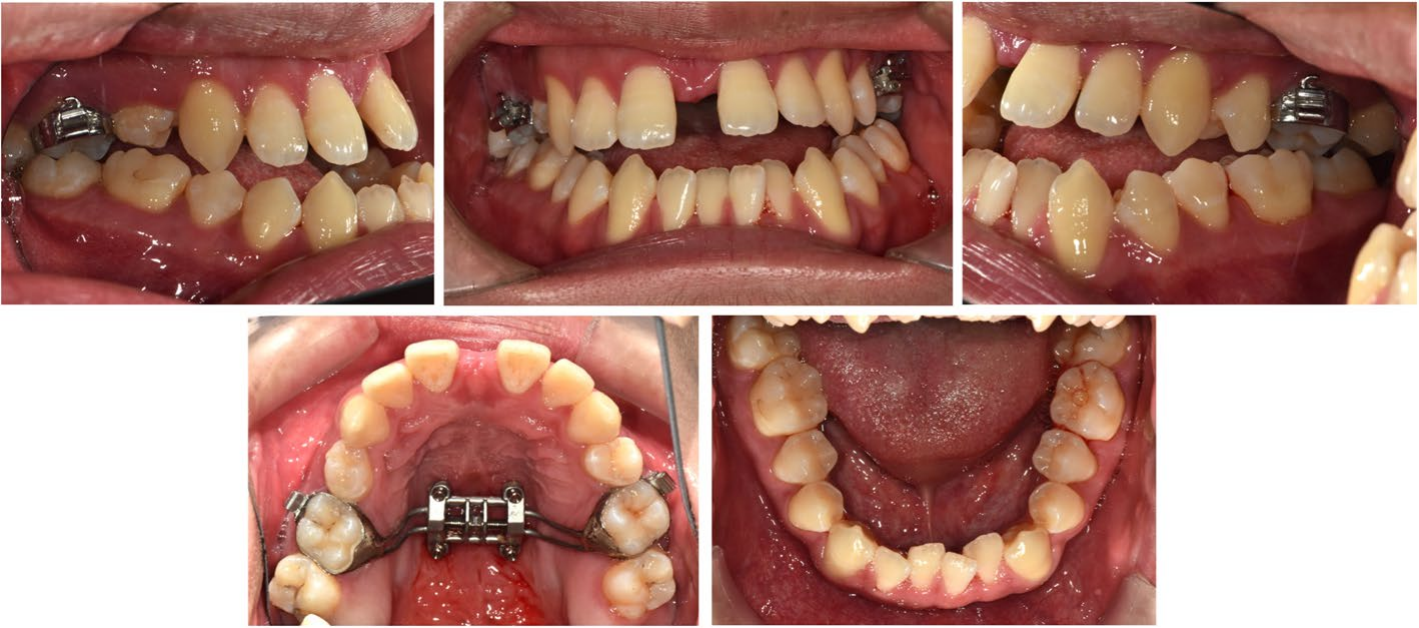
Post-expansion intraoral photos. (47 turns of the expander) Buccal tipping of the molars was a frequent side effect of MSE treating adult patients. The molar bands were then removed to allow dental relapse, leaving only the central MSE-screw combination part to secure sutural bone consolidation

After insertion of MSE, a semi-rapid expansion protocol of one turn per day was instructed [31, 32]. In six weeks, the required expansion was achieved, and the bilateral palatal crossbites were corrected (Fig. 11).

**Fig. 12:**
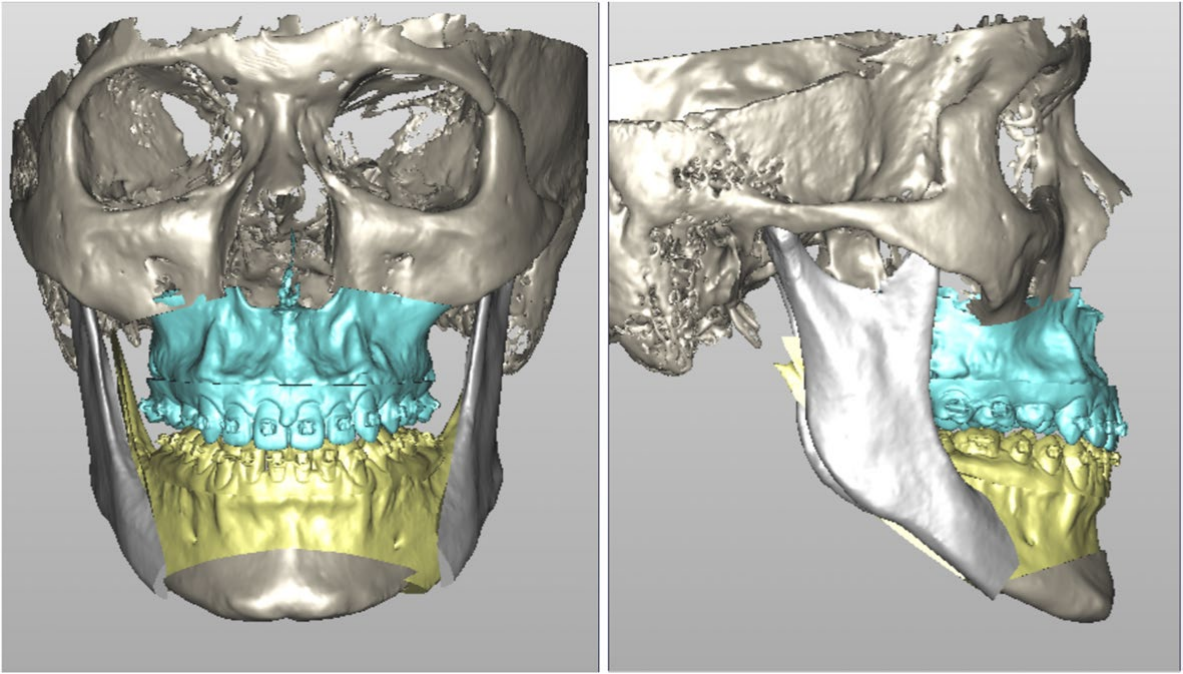
Simulation of Orthognathic surgery. Because the transverse discrepancy was corrected with the MSE in the pre-surgical orthodontic treatment, one-piece maxilla movement was planned

One month after the successful maxillary skeletal expansion, banding and bonding were arranged for leveling and alignment. The maxillary midline diastema started to close in the third month after the cessation of expansion. The MSE was left in place for six months for retention. After eight months of presurgical orthodontic decompensation, two-jaw surgery was performed: maxillary one-piece advancement and mandibular setback with rigid fixations. A genioplasty was performed to reduce chin length and correct asymmetry further (Fig. 12).

**Fig. 13:**
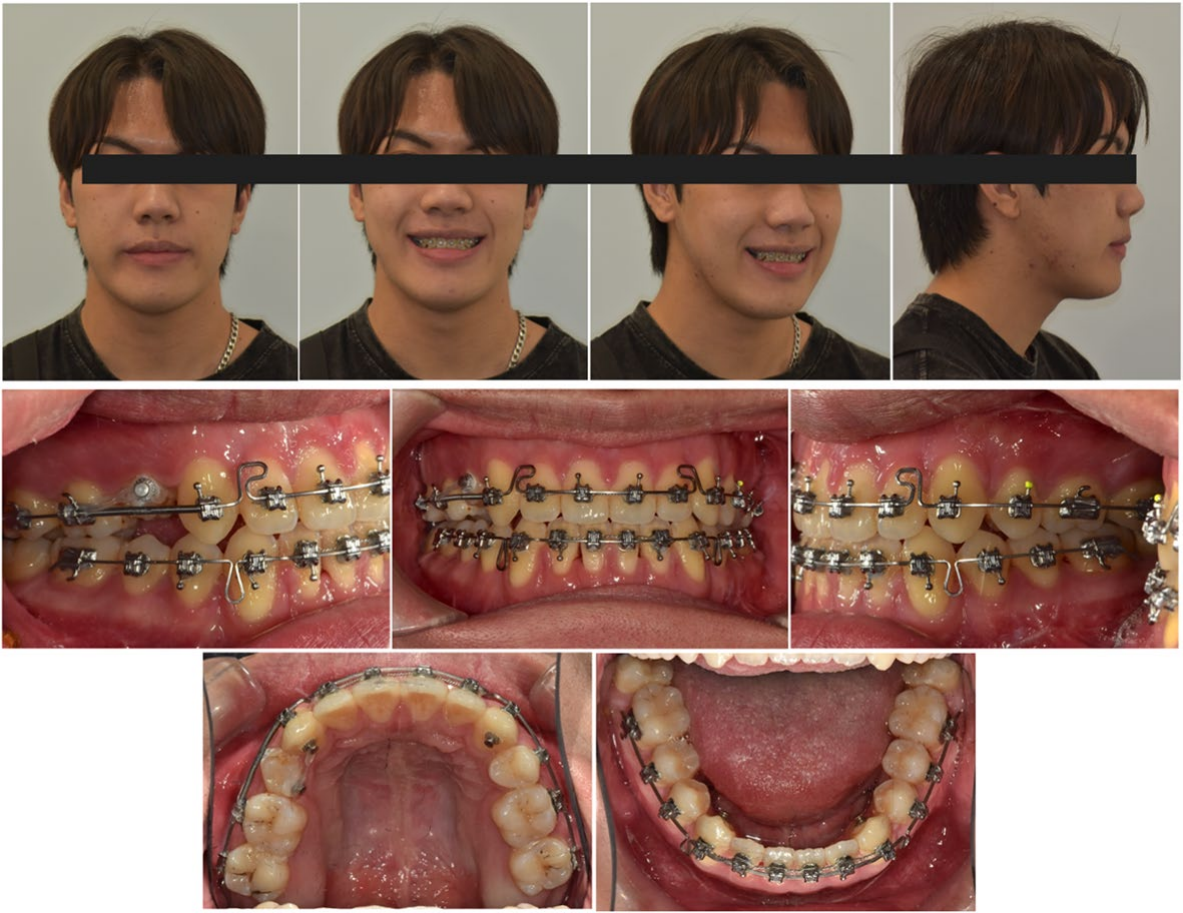
Post-surgical extraoral and intraoral photos after two-jaw orthognathic surgery after 1 year and 8 months of treatment. Mandibular prognathism and facial asymmetry were largely improved with a balanced lateral profile. The molars were relapsed to its physiological position, and the periodontal status remained unchanged

**Fig. 14:**
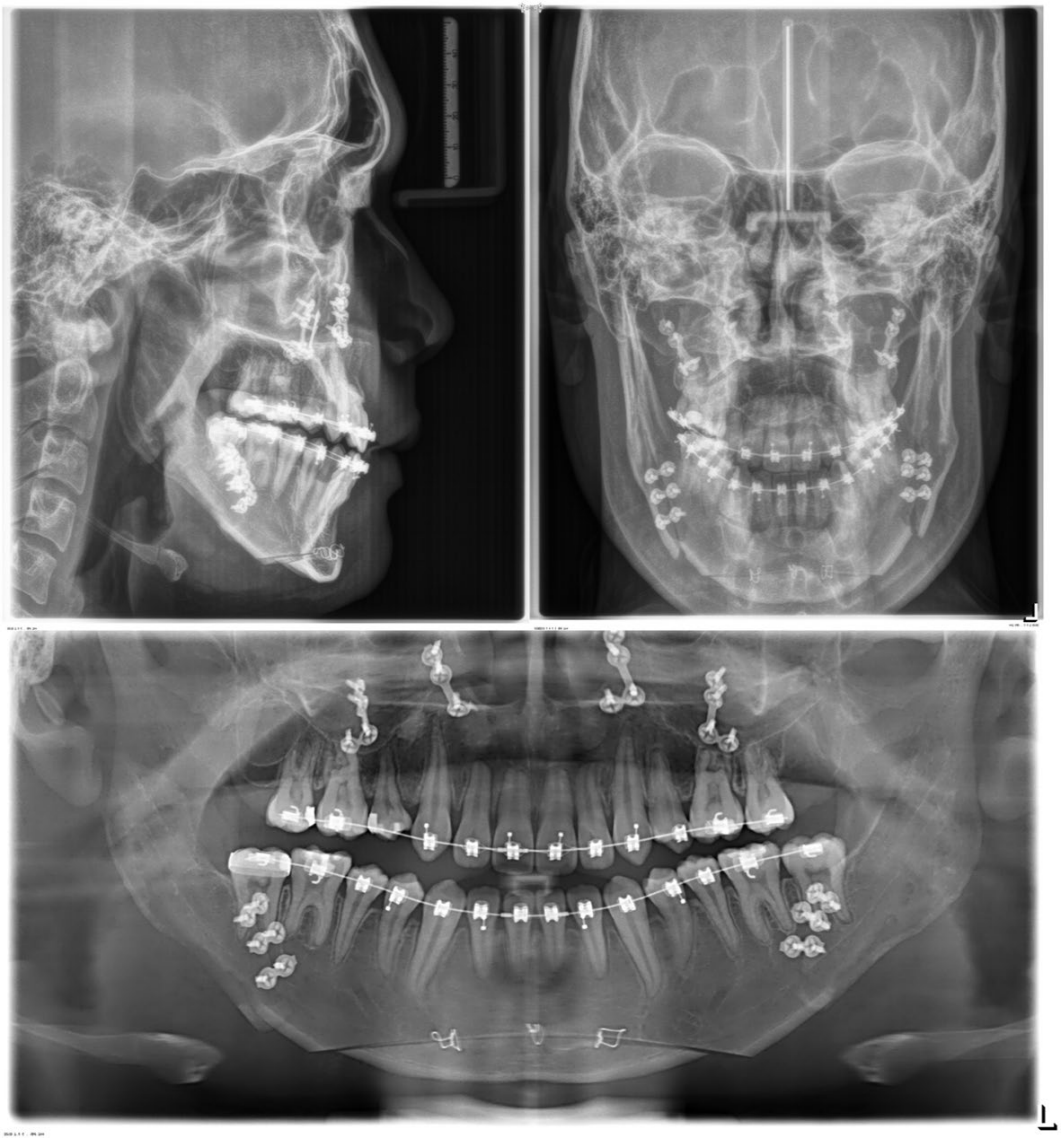
Post-surgical radiographs after two-jaw orthognathic surgery

The patient showed significant improvement in his facial esthetics after the surgery. The bilateral facial volume became more symmetric, occlusal canting was corrected, and his profile became more pleasant. Intraorally, his transverse discrepancy was eliminated, and his periodontal status was well-maintained. The patient continued to be seen monthly for post-surgical detailing (Figs. 13, 14).

## Discussion and conclusions

Several paradigm-shifting inventions and treatment modalities in the orthodontic field have changed the envelope of tooth movement as well as the decision-making process, including the application of temporary anchorage devices, periodontally accelerated osteogenic orthodontics or phenotype modification therapy, and mini-implant assisted rapid maxillary expansion. Before the pioneering report of Lee [15], treating adult patients with transverse skeletal problems required surgical aid, which increased cost and patient morbidity. After MARPE became a generally-accepted approach, cases with maxillary transverse deficiency or borderline Class III cases could be resolved with non-surgical skeletal expansion and maxillary protraction in adolescent and young adult patients [15, 17, 33–36].

**Fig. 15:**
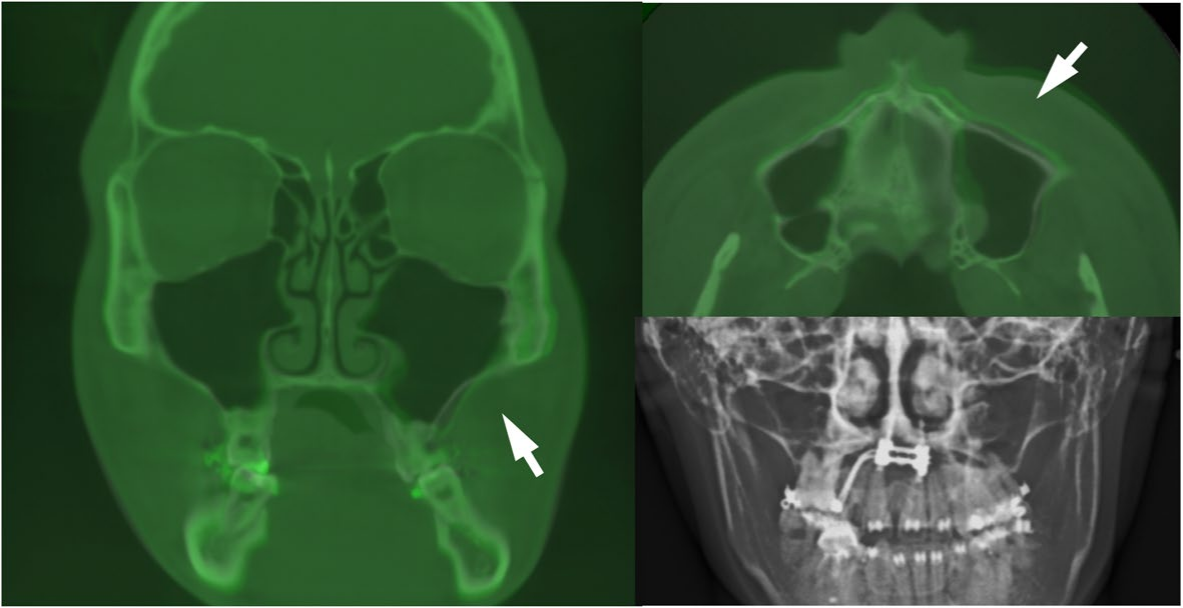
This is another case with an unfavorable expansion result using the traditional MSE fabrication process: unusual expansion pattern (white arrow) revealed by CBCT superimposition (white line, initial; green line, post-expansion). The canted palatal plane caused the right TADs inclination toward the maxillary suture during inserting MSE. The appliance in this case was thus removed to allow relapse of the dentoalveolar tissue

**Fig. 16:**
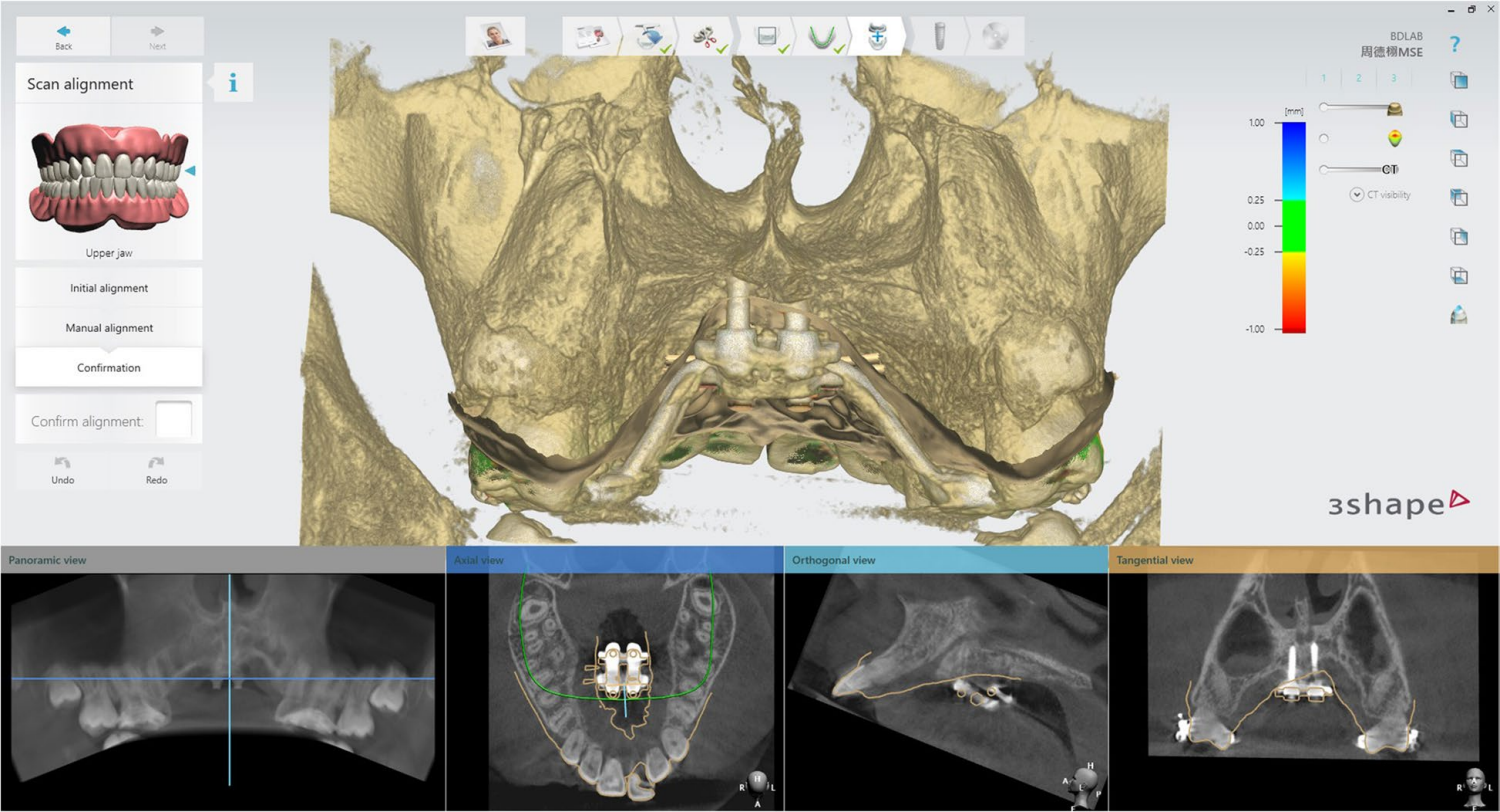
In another case with canted palatal plane, using digital workflow can secure the MSE in a correct position for intended force vector, avoid being mislead by the shape of palate

Previous researchers have investigated key factors affecting the success rate of MSE, including the appliance itself, the anteroposterior position of mini-implants, the bi-cortical vs. mono-cortical penetration of mini-implants, bone thickness, sex, chronological age, and activation frequency, all of which affect the final result of expansion [26, 37, 38]. Among them, the bone thickness or density and the gender or age of a patient cannot be controlled. Consequently, the crucial element is how well we control the remaining factors, and the positioning of the appliance is one of the most important factors in achieving a successful expansion. The term MARPE (mini-implants assisted rapid palatal expander) is loosely used for any expansion device that incorporates mini-implants. Among the countless number of MARPE designs, they can be divided into two categories from the perspective of implant placements. In the early days of MARPE development, most appliances were bone-driven, meaning the mini-implants were placed where a large quantity of bone was present. These MARPEs favored the anterior and lateral walls of the palate, which produced more anterior and inferior expansion [22, 23]. The MSE was a unique MARPE that was a resistant-driven system, meaning the mini-implant position was designed to overcome the resistance against the expansion. The resistance against expansion, not only comes from interlocked midpalatal suture, but also from the zygomatic buttress bones, interlocked pterygopalatine sutures, and other peri-maxillary sutures [39, 40]. Most of the resisting structures mentioned above are in the posterior aspect of the maxilla, and a posterior force vector is required to overcome the resistance. The MSE appliance should be positioned in the posterior palate between the right and left zygomatic buttress bones [28, 30]. However, the posterior palate has a relatively thin bone, and the mini-implants must be placed in a secure area, immediately lateral to the midpalatal suture where the bone has a higher density and greater thickness [25]. The min-implants must be engaged bicortically to ensure the stability [26]. Because of individual variation in anatomical structures, the positioning of the appliance must be decided with several factors in mind: position of the zygoma, septal configuration, midpalatal suture orientation, palatal inclination, canting of the palatal vault, and thickness of the palatal bone. In the past, MSE was fabricated by positioning the appliance in the posterior palate with care and consideration of the above factors. With the advent of recent digital technology and CBCT acquisition, much more precise measures can be taken to locate an optimal MSE position. This report offers a fully digitized solution for MSE positioning to increase the success rate and avoid unwanted expansion results (Figs. 15, 16).

Commercially available MARPE appliances often combine two features in one: an expander and a surgical stent. The expander provides the line of force action, while the surgical stent aids in inserting the mini-implants in the desired location. To identify the best position for both parts, several digital workflows have been proposed by previous researchers using various expander designs [28–30, 41]. Often these systems require a two-step process: mini-implant insertion by a digitally produced stent and retrofitting of MARPE onto the mini-implants by digital processing. The surgical stent is not required for MSE since the appliance comes with guiding slots for the mini-implants, and the appliance itself serves as a surgical guide, eliminating the retrofitting of the appliance to the mini-implants. An optimal MSE position, based on the desired force vector, determines the mini-implant position without the two-step procedure. Consequently, finding an optimal MSE position is extremely important. Cantarella et al. combined a segmented maxillary bony structure from CBCT and a dental model surface from an intraoral scanner, placed a digital MSE template in an optimal position based on CBCT information, and then digitally designed a position guide to replicate the same MSE position on the stone model. Lo Giudice et al. developed a similar method to locate a negative template of the MSE device on the digital model and printed it as a reference for band soldering. Both approaches embraced the idea of digital design, but the final process of band soldering was not incorporated, resulting in an extra appointment for the pick-up impression. In this study, upper first molar tooth dies were fabricated and printed digitally with a parallel path of insertion so that the technician could choose an accurate band size and solder the MSE device accordingly. Loose bands often result in an increased caries rate, breakage of the appliance, cement loss, or detachment from the tooth, and this digital approach also facilitates the accuracy of the band size and fitting [42–44].

This report describes a completely digital process that can produce a final soldered appliance using only CBCT and a digital dental model. It is also the first study to describe how band selection can be determined through commercially available software. Although digital workflows could improve patient comfort and reduce appliance fabrication errors that could result in unwanted outcomes, further studies comparing the manual and digital methods in MSE fabrication are still needed to verify the reliability and effectiveness of this method.

The digitally designed MSE workflow, using commercially available software, can reduce patient appointments, shorten the clinical chair time, and prevent adverse events associated with poor design and faulty placement of the appliance. Further studies comparing the MSE fabrication processes and their impacts on clinical outcomes should be conducted to verify the clinical significance.

## Data Availability

The datasets used and/or analysed during the current study available from the corresponding author on reasonable request.
